# Validation of a brief sex addiction screening instrument (PATHOS) and prediction of sex addiction in the Iranian population

**DOI:** 10.47626/2237-6089-2019-0088

**Published:** 2021-02-26

**Authors:** Armin Zareiyan, Hamid Sharif Nia, Nader Molavi, Abdolhadi Saeidi, Mohamadreza Najarzadegan, Masumeh Ghazanfarpour, Hamed Jafarpour, Masoudeh Babakhanian

**Affiliations:** 1 Department of Health Disaster and Emergencies Nursing Faculty Aja University of Medical Sciences Tehran Iran Department of Health Disaster and Emergencies , Nursing Faculty , Aja University of Medical Sciences , Tehran , Iran .; 2 Department of Nursing Mazandaran University of Medical Science Sari Iran Department of Nursing , Mazandaran University of Medical Science , Sari , Iran .; 3 Kashan University of Medical Sciences Kashan Iran Kashan University of Medical Sciences , Kashan , Iran .; 4 Urmia University of Medical Sciences Urmia Iran Urmia University of Medical Sciences , Urmia , Iran .; 5 Research Center for Addiction and Risky Behaviors Iran University of Medical Sciences Tehran Iran Research Center for Addiction and Risky Behaviors , Iran University of Medical Sciences , Tehran , Iran .; 6 Student Research Committee Kerman University of Medical Sciences Kerman Iran Student Research Committee , Kerman University of Medical Sciences , Kerman , Iran .; 7 Psychiatry and Behavioral Research Centre Addiction Institute Mazandaran University of Medical Sciences Sari Iran Student Research Committee, Psychiatry and Behavioral Research Centre , Addiction Institute , Mazandaran University of Medical Sciences , Sari , Iran .; 8 Semnan University of Medical Sciences and Health Services Semnan Iran Semnan University of Medical Sciences and Health Services , Semnan , Iran .

**Keywords:** Sex addiction, reliability, validity, Iran

## Abstract

**Introduction:**

Sex addiction is a major psychiatric disorder in which a person is compelled to participate in sexual activities despite negative consequences. This study was conducted to localize a brief sex addiction screening instrument (PATHOS) for use in the Iranian population and to determine variables predictive of sex addiction in the general Iranian population.

**Methods:**

In this study, we evaluated the psychometric properties of PATHOS in a sample of 443 Iranians in 2018. Data were analyzed using exploratory factor analysis (Factor 10.8.04 software). Simple and multiple linear regression analyses were used to investigate construct validity and variables predictive of addiction.

**Results:**

Exploratory factor analysis identified two factors in this dichotomous questionnaire and reported the questionnaire’s test-retest reliability in the target population. Prognostic variables for sexual addiction in the Iranian population were determined to be female gender, higher education, viewing pornographic videos, having multiple sex partners, having difficulty interacting in sex, and history of masturbation.

**Conclusion:**

The Persian version of the brief sex addiction screening instrument (PATHOS) has sufficient reliability and validity in the Iranian population. The predictive variables of sex addiction are indicative of the presence of risk of this disorder in Iranian samples and more studies are needed in order to enable prevention and treatment.

## Introduction

Sex addiction is a major psychiatric disorder in which a person has a compulsion to participate in sexual activity despite negative consequences. ^1^ The term sex addiction is used exclusively for those who report not being able to control their own sexual urges, behaviors, or thoughts. ^2^ Sexual hyperactivity disorders include a variety of behavioral problems such as excessive masturbation, cybersex, pornography, phone sex, visiting strip clubs, and sexual activity with consenting adults. Consequences of engaging in these behaviors are similar to those of other addictive disorders. ^3 , 4^

The concept of sex addiction is controversial among professionals, and currently there is no section for diagnosis of compulsive sexual behavior, hypersexual disorder, or sex addiction in Diagnostic and Statistical Manual of Mental Disorders, 5th edition (DSM-5). However, the International Classification of Diseases, 11th revision (ICD-11) does contain a new classification of compulsive sexual behavior disorder in the impulse control disorder section: “a persistent pattern of failure to control intense, repetitive sexual impulses or urges, resulting in repetitive sexual behavior over an extended period (e.g., six months or more) that causes marked distress or impairment in personal, family, social, educational, occupational, or other important areas of functioning.” ^5^

The prevalence of compulsive sexual behavior disorder varies from 3 to 6% in adult populations, ^4 , 6^ but a recent study reported it at 1 to 3%. ^7^ Carnes also claims about 50% of sex offenders fulfill the criteria for sex addiction. ^8^ In general, since people have become more likely to use smart phones, tablets, laptops, and free Wi-Fi, it seems their participation in online sexual behaviors has increased. ^9^ However, due to disparate assessment tools all over the world, the available research and evidence on the prevalence of this disorder in different cultures is limited, and data on those affected who have never received any therapy is lacking. ^3^

In recent years, many studies have been conducted on the diagnosis, evaluation, and treatment of sex addiction. ^1^ Two other available screening tests are the Sexual Addiction Screening Test (SAST) and the Compulsive Sexual Behavior Inventory (CSBI). The SAST measures four core items of preoccupation, loss of control, relationship disturbance, and affect disturbance using 25 self-report yes/no items and can be used to assess sexually compulsive behavior and, as a result, presence of sex addiction. SAST results help to differentiate between addictive and non-addictive behaviors. ^10^ The CSBI employs 28 self-report items to evaluate compulsive aspects of sexual behaviors in three subscales of sexual impulse control (paraphilic versus nonparaphilic), abuse, and violence. ^11^ In this study we used the PATHOS screening tool.

The PATHOS questionnaire is a short version of the Sexual Dysfunction Assessment that measures six signs of sex addiction and was developed by Carnes et al. in 2012. ^2^ In 2015, this tool was standardized by Cashwell et al. with an undergraduate student population, ^3^ but the results demonstrated low internal consistency among item scores. ^3^ So far, this questionnaire and a similar version of it called BODIES have been standardized with college students in North Carolina. ^3 , 4^

Use of online pornography is on the rise in the world, and because of its wide availability, the ability to access it anonymously, and its affordability, ^5^ it is becoming a major behavioral addiction in Iran. To the best of our knowledge, no tools have yet been developed for measuring sex addiction among clinical and general Iranian populations. This study was conducted in order to localize the first tool for sex addiction assessment, entitled PATHOS, in the general population of Iran.

## Material and method

### Measure: A brief screening instrument for assessing sexual addiction (PATHOS)

This short, six-question tool was first developed by Carnes et al. in 2012 in order to evaluate and screen healthy and sick people for sex addiction. The items in this tool were extracted from the Sexual Addiction Screening Test (SAST). The answers to the questions in PATHOS are dichotomous (yes/no). The cut-off point for this questionnaire is 3, which is used to distinguish healthy subjects from patients. The tool has one factor and can be administered in less than one minute and can therefore help physicians to screen individuals in clinical settings. ^2^ The six items are as follows:

1. Do you often find yourself preoccupied with sexual thoughts? [ **P** reoccupied]
2. Do you hide some of your sexual behavior from others? [ **A** shamed]
3. Have you ever sought help for sexual behavior you did not like? [ **T** reatment]
4. Has anyone been hurt emotionally because of your sexual behavior? [ **H** urt others]
5. Do you feel controlled by your sexual desire? [ **O** ut of control]
6. When you have sex, do you feel depressed afterwards? [ **S** ad]

### Procedure

This is a psychometric study; its statistical population encompasses all individuals living in Iran. The Student Research Committee of Mazandaran University of Medical Sciences provided financial support (project 175; accepted June 30, 2017) and granted ethics approval (IR.MAZUMS.REC.1396.175) for this study.

Monroe believes the optimum sample size for a factor analysis is 200 to 500. ^6^ Since a large number of respondents accessed our questionnaire online through the Telegram application, the number of people who viewed the questionnaire is not available. Evidence suggests adequate response rate to an online form is 50%. ^7^ Since a sufficient sample size for factor analysis is between 200 and 500, ^6^ the sample size of the current study, at 443 participants, is considered to be sufficient. The results of this study were analyzed in two steps.

First, in order to study the psychometric properties of PATHOS, its items were first translated into Farsi by two experts fluent in both English and Farsi. Using content validity index (CVR) and content validity ratio (CVI), the content validity indexes for all items were found to be acceptable. No questions were removed or changed. The Farsi version was then back-translated into English and sent to the original author for verification. Finally, the questionnaire was administered, and the data collected and analyzed. In order to evaluate the tool’s test-retest reliability, we calculated intra-class coefficient correlations (ICC) for 14 participants who were retested after nine days, using the Statistical Package for the Social Sciences (SPSS) version 18 (ICC: 0.916 [95% confidence interval {95%CI} 0.73-0.97]; Sig = 0.000), which indicated that the tool had favorable stability. Factor 10.8.04 software was used to confirm construct validity. We employed exploratory factor analysis to investigate the appropriateness of inferences made based on the tool and to determine construct validity for the questionnaire in the Iranian society. The total number of participants in this study was 443 and the tool has 6 items, all of which were included in the study. Because of the dichotomous nature of the responses, the polychoric correlations matrix was used. The data extraction method used was robust unweighted least squares (RULS); an exploratory factor analysis method. The factorial rotational method used was direct oblique rotation. Factor 10.8.04 software was used for the factor analysis.

To identify variables predictive of sexual addiction in the Iranian samples, we described the demographics of the participants using descriptive statistics and used the Kolmogorov-Smirnov test to verify normal distribution of the semi-continuous data. Backward stepwise regression was employed to identify predictors of sexual addiction. The Durbin-Watson test was used to investigate the assumption of non-correlation of residuals (independent errors in linear regression). Variance inflation factor (VIF) and tolerance were used to investigate the assumption of multicollinearity non-correlation. The significance level adopted in the linear regression model was p < 0.05. The Statistical Package for the Social Sciences (SPSS) version 18 was used to analyze the data.

### Participants

The inclusion criteria for this study were: Iranian nationality, age under sixty, and ability to read and write. The study questionnaire was hosted online and sent anonymously to the participants via email or the Telegram messaging application. Additionally, a number of questionnaires were distributed to university students. Before opening the link to the questionnaire, the research goals were explained to the participants and they were instructed to continue only if they consented to participation in the study. Incompletely answered questionnaires were excluded.

## Results

### Demographics

Of 443 participants, 228 (51.5%) were female and 215 (48.5%) were male. The most prevalent age group was 18 to 29 years. The substances most abused by participants were nicotine and alcohol (93; 21%). About 59.4% of the respondents had a score of three points or fewer and were categorized as normal, in accordance with the clinical cut-off point for the original tool. The remaining 40.6% scored more than three points and potentially had sex addiction, needing further investigation and possibly treatment in clinical settings. Tables 1 and 2 show the participants’ demographics and their sexual history.

**Table 1:** Demographic characteristics of the study participants

Characteristics	Frequency (%)
Age (years)	
> 18	7 (1.6)
18-29	217 (49.0)
30-39	106 (23.9)
40-49	41 (9.3)
< 50	18 (4.1)
Ethnicity	
Fars	163 (36.8)
Kord	55 (12.4)
Lor	22 (5.0)
Turk	75 (16.9)
Other (Turkman, Gil, Mazani, Arab, Baluch)	128 (28.9)
Educational level	
Up to high school diploma	88 (19.8)
Four-year university degree	191 (43.1)
Postgraduate education	164 (37.1)
Marital status	
Married	166 (38)
Single	221 (50
Divorced or widowed	53 (12
Residence	
With family	345 (77.8)
With roommate	43 (9.7)
Alone	52 (11.8)
With partner	3 (0.7)
Religion	
Muslim	359 (81)
Other religions (Zoroastrian, Jewish, Christian)	8 (1.8)
No religion	76 (17.2)

**Table 2:** Sexual history characteristics and psychiatric disorders of the study participants

Variable	n (%)
Thinking about, and focusing on, sexual matters all the time	
Yes	38 (8.6)
No	82 (18.5)
Somewhat	323 (72.9)
Sexually transmitted infections (HIV, HCV, HPV)	
Yes	38 (8.6)
No	405 (91.4)
Multiple sexual partners outside wedlock	
No	302 (67.7)
Yes	141 (32.3)
Sexual orientation	
Same sex	19 (4.3)
Opposite sex	404 (91.2)
Both	20 (4.5)
Any lifetime drug use	
Yes	120 (27.1)
No	323 (72.9)

### Validity, reliability, and exploratory factor analysis

We used the Kuder-Richardson coefficient to evaluate internal consistency. The value was 0.91, which is acceptable. For test-retest repeatability, we calculated the intra-class correlation coefficient (ICC) for 14 participants retested after nine days (ICC: 0.916 [95%CI: 0.73-0.97] Sig=0.000) which proved the questionnaire is highly reliable. The first step in exploratory factor analysis was to verify the tool’s subscales or, in other words, the validity of the construct in Iranian society was confirmed. The total number of participants in this study was 443 and the total number of variables was six, all of which were included in the study. A polychoric correlation matrix was used. The robust unweighted least squares (RULS) exploratory factor analysis method was used for data extraction. Oblique rotation was then used to determine factor correlations. First, we present a descriptive explanation of the variables in Table 3 .

**Table 3:** Univariate descriptive of variables

	Variable	Mean	95%CI	Variance	Skewness	Kurtosis (zero centered)
1	Sexual treatment	0.099	0.06-0.14	0.089	2.685	5.197
2	Sexual control	0.81	0.76-0.86	0.154	-1.587	0.516
3	Sexual preoccupation	0.413	0.35-0.47	0.242	0.354	-1.873
4	Sexual guilt-sadness	0.718	0.66-0.77	0.203	-0.970	-1.059
5	Sexual worry-shame	0.772	0.72- 0.82	0.176	-1.300	-0.312
6	Sexual family problems-hurt others	0.269	0.21-0.32	0.196	1.046	-0.905

95%CI = 95% confidence interval.

Parallel analysis was used to determine the number of factors, identifying two factors. As shown in Table 4 , together these factors explained about 0.73 of the total variance, which is an acceptable number.

**Table 4:** Explained variance, according to eigenvalues

Variable	Eigenvalue	Proportion of variance	Cumulative proportion of variance explained
1	3.25988	0.54331	0.54331
2	1.17453	0.19575	0.73907
3	0.72795	0.12133	
4	0.54295	0.09049	
5	0.20370	0.03395	
6	0.09099	0.01517	

Using variable factor loading, the results proved two factors were involved. In item five, the values for both factors were more than 0.3. Since the difference between these two factors was more than 0.2, the larger value (factor 1: 0.628) was reported.

The results of the exploratory factor analysis showed that this tool reports two factors in the Iranian population: psychosocial problem (sexual treatment, sexual control, sexual guilt-sadness, sexual worry-shame) and craving (sexual preoccupation and sexual family problems-hurt others).

### Regression models predicting sexual addiction

Univariate linear regression analysis demonstrated that being female, higher educational level, unit increase in time spent watching pornography, history of difficult sexual encounters, having multiple sexual partners, and history of masturbation could meaningfully predict sexual addiction scores in the Iranian population. Multivariate linear regression analysis then showed that only variables that were retained in the backward model predicted rates of addiction in the Iranian population (each unit increase in education level, history of difficult sexual encounters, and history of masturbation), explaining 47.3 percent of variance in rates of addiction.

## Discussion

The first part of this study focuses on validation of the Iranian version of the PATHOS questionnaire with a non-clinical sample from the general population. Based on the results, the Farsi version of the PATHOS questionnaire has sufficient validity for Iranian samples. The results of exploratory factor analysis showed that this tool, when used for the Iranian population, has two subscales: psychosocial problems and craving; items in these subscales assess related issues.

One of the main limitations of Carnes’ original tool is the failure to report evidence on factors, ^2^ which has been corrected in our study. The factors explained about 0.73 of total variance, which is an acceptable number. Another limitation of the original study is that temporal stability was not reported. ^2^ In order to eliminate this limitation, 14 participants were retested after nine days; the test-retest results indicated high correlation.

In similar studies, validation was carried out on specific populations, ^2 , 3^ but in our study the tool was administered to the general population, and it is safe to say it can be used for screening in the general population. However, our study has limitations. First, the questionnaire was administered as a self-report tool (for non-clinical use) to examine the sexual behaviors of a population who were fully aware of their behaviors and attitudes toward sex. Second, the online survey method implies that participants entered the study through a self-selection recruitment procedure, which can affect results, and therefore the results should be interpreted with caution. ^12^ Third, based on the clinical cut-off point of the original tool, the estimated prevalence of sex addiction in our study was about 40% higher than the comparable prevalence in similar studies, ^13 , 14^ probably due to participation bias: more people with higher levels of concern about sexual addiction opted to participate in this study. Fourth, for future studies in Iran, it is recommended that the tool be administered in conjunction with a clinical interview in order to determine a clinical cutoff point for those addicted to compulsive sexual behaviors.

The second part of this study was designed to identify variables predictive of sexual addiction in Iranian samples. The results of multiple linear regression analysis showed that female gender, higher education, history of watching pornography, multiple sexual partners, history of difficult sexual encounters, and a history of masturbation were the most important predictors of sexual addiction in this Iranian population. Similar studies have also shown a positive correlation between higher education and sex addiction. ^15 , 16^ Achieving higher education probably provides higher social status and, as a result, better sexual opportunities. ^16^

The second part of this study has some limitations; the most important of which is online administration of the questionnaire, which provides a self-reported temporal cross-section of the participants’ state of mind, and is therefore prone to social desirability bias. ^17^ Future studies should also measure the role of craving and compulsion in using cybersex as an important predictor of sex addiction. ^18^

Studies show that sex, like other addictive behaviors, involves the reward system in the brain, ^19^ and sexual self-gratification behaviors (such as fantasies, pornography, intercourse, or masturbation) are potentially addictive. ^20 , 21^ Therefore, in those who engage in excessive sexual gratification, sexual impulses may become uncontrollable and cause them to spend a lot of time thinking about sex and participating in sexual activities. ^22^

Iran’s laws are based on Islamic teachings. In order to avoid prosecution, people often engage in sexual behaviors surreptitiously, which may hinder pursuit of healthy sexual interactions. ^23^ Traditional parenting and Iran’s orthodox educational system contribute to a lack of basic training in control of sexual behavior and regulation of emotional excitement, causing difficulties in sexual interaction between individuals. ^24^ Excessive involvement in sexual activities is correlated with mood and emotion dysregulation (depletion of thoughts and feelings) and seems to be an important predictor of persistent addictive sexual behaviors and difficulty with interpersonal interactions. ^21^

Since the advent of the Internet, most people spend part of their lives online. Studies show that those who engage in online sexual behaviors watch pornography an average of 15 to 25 hours a week. ^25^ The results also show gender difference has an important role in the incidence of addictive sexual behaviors. ^21^ The results of our study showed that, in Iran, female gender is an important predictive variable of sex addiction incidence. Studies show women are more likely to engage in online sexual activities such as sex chat in chat rooms. ^13^ Sensing a threat to personal identity, dissatisfaction with gender identity, and having painful emotional experiences, such as infidelity, are important factors threatening the sexual identity of Iranian women and predisposing them to addictive sexual behaviors. ^22 , 26^

## Conclusion

This is the first study to localize a sexual addiction measurement tool for Iran. The results showed that the Farsi version of the brief sex addiction screening instrument (PATHOS) has sufficient reliability and validity for use in Iranian populations. Also, according to demographic variables, Iranian women are more susceptible to this disorder. Other personal variables important for prediction of sex addiction risk were also identified, explaining 47% of variance. More studies are needed for prevention and treatment of this disorder.

**Table 5:** Exploratory factor analysis with Varimax rotation

	Variable	Communality of rotated loading matrix	Rotated loading matrix	
			Factor 1 (Psychosocial problem)	Factor 2 (Craving)
1	Sexual treatment	0.244	0.530	
2	Sexual control	0.742	0.715	
3	Sexual preoccupation	0.452		0.658
4	Sexual guilt-sadness	1.000	0.960	
5	Sexual worry-shame	0.683	0.628	
6	Sexual family problems-hurt others	0.504		0.711
ORION (8, 9)			0.997	0.718

**Table 6:** Simple linear regression analysis and multiple linear regression analysis to predict sex addiction variables in an Iranian population.

Variable	B	95%CI
Univariate linear regression analysis results		
Female gender	-0.515	-0.793 to -0.237
Educational level	-0.211	-0.329 to -0.092
Watching pornographic videos	0.288	-0.476 to 0.100
History of difficult sexual encounters	2.085	1.864 to 2.364
Having multiple sexual partners	0.329	0.035 to 0.624
History of masturbation	0.635	0.201 to 1.070
Multivariate linear regression analysis results		
Educational level	-0.096	-0.190 to -0.002
History of difficult sexual encounters	2.000	1.775 to 1.225
History of masturbation	0.402	0.670 to 0.737
Adjusted R ^2^ = 0.473		

p < 0.05.
